# Ethanol embolization of arteriovenous malformations in the buttock: ten-year experiences in diagnoses and treatment options

**DOI:** 10.1186/s13023-024-03205-x

**Published:** 2024-05-13

**Authors:** Yuchen Shen, Deming Wang, Xindong Fan, Lianzhou Zheng, Lixin Su, Xitao Yang

**Affiliations:** 1 Vascular Anomaly Center, Department of Interventional Therapy, Shanghai Ninth People’s Hospital, Shanghai Jiao Tong University School of Medicine, No. 639, Zhi Zao Ju Rd, Shanghai, 200011 China; 2 Fengcheng Hospital of Feng Xian District, Fengcheng Branch, Shanghai Ninth People’s Hospital, Shanghai Jiao Tong University School of Medicine, Shanghai, 201413 China

**Keywords:** Arteriovenous malformations, Buttock, Ethanol, Embolization, Outcome

## Abstract

**Background:**

Clinically, arteriovenous malformations in the buttocks (bAVMs) are extremely rare. Our study aimed to evaluate the efficacy and safety of ethanol embolotherapy in managing bAVMs.

**Results:**

A total of 32 patients with bAVMs (14 females and 18 males) from 2012 to 2021 were included in this study. All patients underwent complete clinical and imaging examinations. Further, the AVMs lesions were analyzed according to Schöbinger staging and Yakes classification. Each patient had undergone a multistage ethanol embolization. The amelioration of clinical symptoms and devascularization on angiography were evaluated at regular follow-ups. In the present cohort, the 11–20 age group had the most patients (15/32; 46.88%). A total of 124 embolization procedures were performed (average 3.88 procedures per patient), and the average dose of absolute ethanol was 18.96 mL per procedure. Thirteen patients with dominant draining veins underwent additional coil deployment before ethanol embolization (13/32; 40.63%). During follow-ups, clinical improvement was found in 23 of 27 who presented with a pulsating mass (85.19%), 17 of 20 with abnormal local skin temperature (85%), 5 of 6 with bleeding (83.33%), and 5 of 5 patients treated for pain (100%). More than 75% angiographic devascularization was achieved in 18 patients (18/32; 56.25%). Finally, 12 out of 13 patients (92.31%) reduced from Schöbinger Stage III to a lower grade, and ten patients exhibited a complete response (10/32; 31.23%). There was a single serious complication of local necrosis, while neither paranesthesia nor infection was observed postoperatively.

**Conclusions:**

Ethanol embolization assisted with coils can treat bAVMs effectively and safely. The Yakes classification contributed to the optimal ethanol embolotherapy of bAVMs.

## Background

Arteriovenous malformations (AVMs) are congenital diseases that usually appear in an insidious form at birth and are easily overlooked during childhood [1]. Several factors, including trauma, infection, and hormonal changes, lead to the expansion and deterioration of the AVMs lesions [2].

Peripheral AVMs have a low prevalence rate, approximately (2.15–6.60) /1,000,000 population, whereas AVMs in the buttock (bAVMs) are even rarer, and their treatment is challenging [1]. Symptoms of bAVMs vary from asymptomatic erythema to a potentially life-threatening hemorrhage and even impaired mobility, causing severe functional disorders [3]. Thus, it is necessary to diagnose and intervene in bAVMs timeously and effectively.

To date, the effectiveness of surgical resection of the lesion with or without preoperative embolization in treating bAVMs has only been reported in sporadic cases [4]. Depending on the stage, surgery is a curative therapy for initial disease, while embolization is preferred in advanced non-resectable disease. Ethanol embolotherapy has been widely used to manage AVMs in the oral and maxillofacial regions [5, 6], the scalp [7], and the limbs [8] with promising outcomes and acceptable complications [9]. High-concentration ethanol (80 ∼ 100%) is capable of exerting denaturation to hemocytes, vascular endothelial cells. This will further lead to the occlusion of blood vessels and inhibit the angiogenesis of AVMs’ vasculature [10]. Currently, abnormal hemodynamics have been proven to play a pivotal role in the progression of AVMs [11]. However, in extremely high-flow AVMs, especially those with exceedingly dilated venous lumen, using ethanol alone is ineffective in achieving cessation of flow through the AVMs. From the perspective of angioarchitecture, coils should be configured in the outflow vein with dilated lumen, known as a venous sac, to slow down the blood flow rates. The use of ethanol, accompanied by coils, can help reduce the flow and potentially cure the advanced peripheral AVMs [12].

Given the scarcity of bAVMs-related research, there is still a lack of consensus on the therapeutic effect of an ethanol-based strategy. Therefore, we retrospectively analyzed a cohort of 32 patients with bAVMs, aiming to evaluate the outcomes of coils-assisted ethanol embolization in treating bAVMs.

## Methods

### Patients

Approval for this retrospective study of medical records, photographs, and radiologic imaging was obtained from the Institutional Review Board (No.SH9H-2019-Q-024). All patients’ consent for participation in the study was waived.

**Fig. 1:**
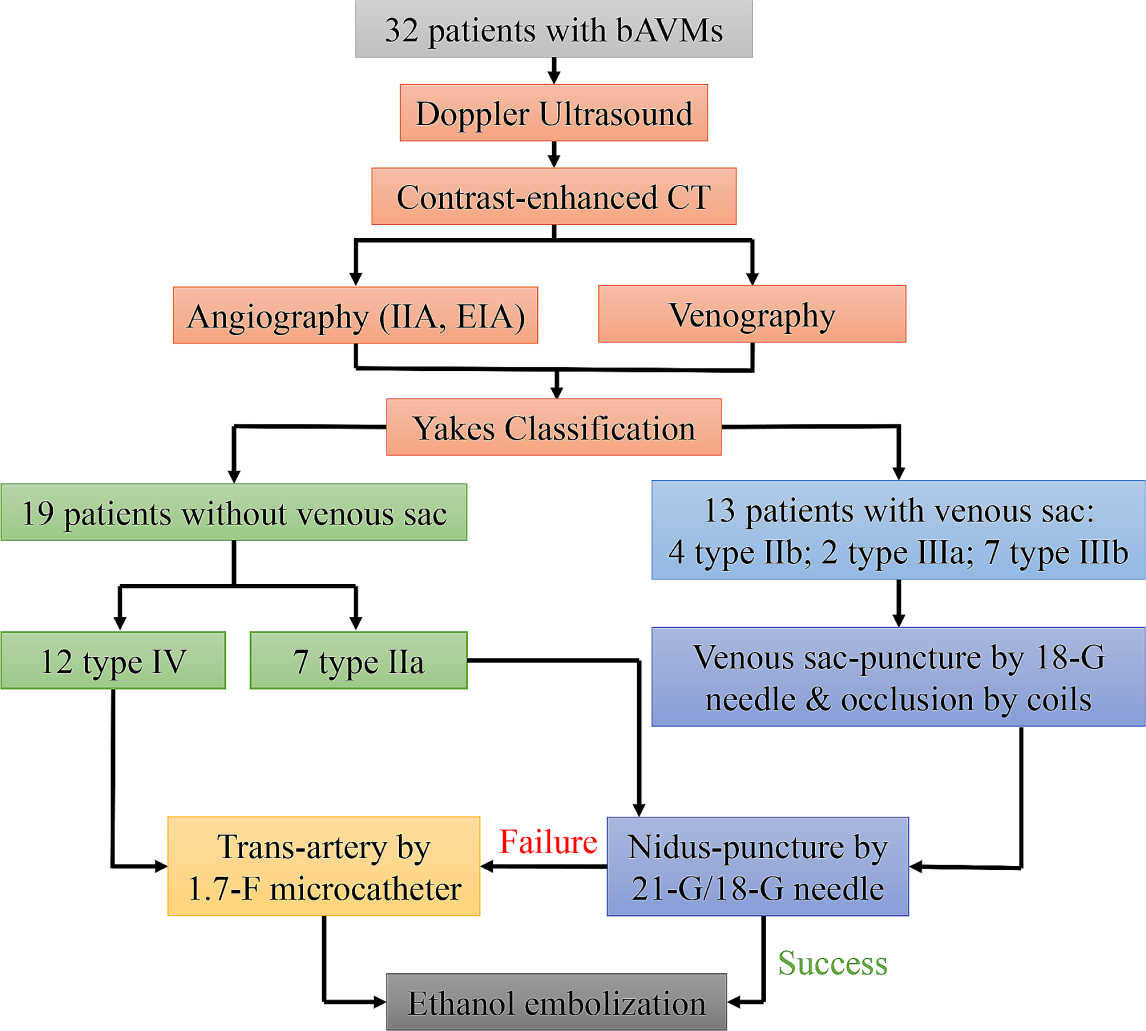
Flow chart of the ethanol embolization of arteriovenous malformations in the buttock (bAVMs). IIA: Internal iliac artery; EIA: External iliac artery

The flow chart of this study is shown in Fig. 1. bAVMs are defined as AVMs that occur in the region of the buttocks, the superior aspect of which ends at the iliac crest, and the lower part is outlined by the horizontal gluteal crease [13]. Physical examination demonstrated common symptoms of bleeding, bloody stool, erythema, elevated skin temperature (measured by the infrared measure temperature gun), pain, pigmentation, pulsating mass, swelling (the inelastic tapeline measured hip perimeter), and ulceration. A total of consecutive 32 patients with bAVMs who had received ethanol embolization treatment in our center from July 25, 2012, to August 1, 2021, were included.

After the clinical investigation, Doppler ultrasound (DUS) and contrast-enhanced computed tomography (CT) were used to evaluate the hemodynamic and anatomic features of the bAVMs. According to Schöbinger stage of AVMs, stage II or higher indicates the need for treatment [2]. Contraindications included the following: previous allergic reactions to ethanol or the contrast medium, previous major complications from ethanol embolization, and severe impairment of hepatic, renal, and cardiopulmonary function.

### Procedures of interventional embolization

All ethanol embolization procedures were done under general anesthesia. Pulmonary arterial pressure (PAP) monitoring through the Swan-Ganz catheter (Edwards Lifesciences, Irvine, CA, USA) was not routinely performed. Eleven patients (11/32; 34.4%) had peri-operative PAP monitoring when the estimated volume of absolute ethanol per procedure was more than 0.5 mL/kg based on the size of the lesion [6]. 

**Fig. 2:**
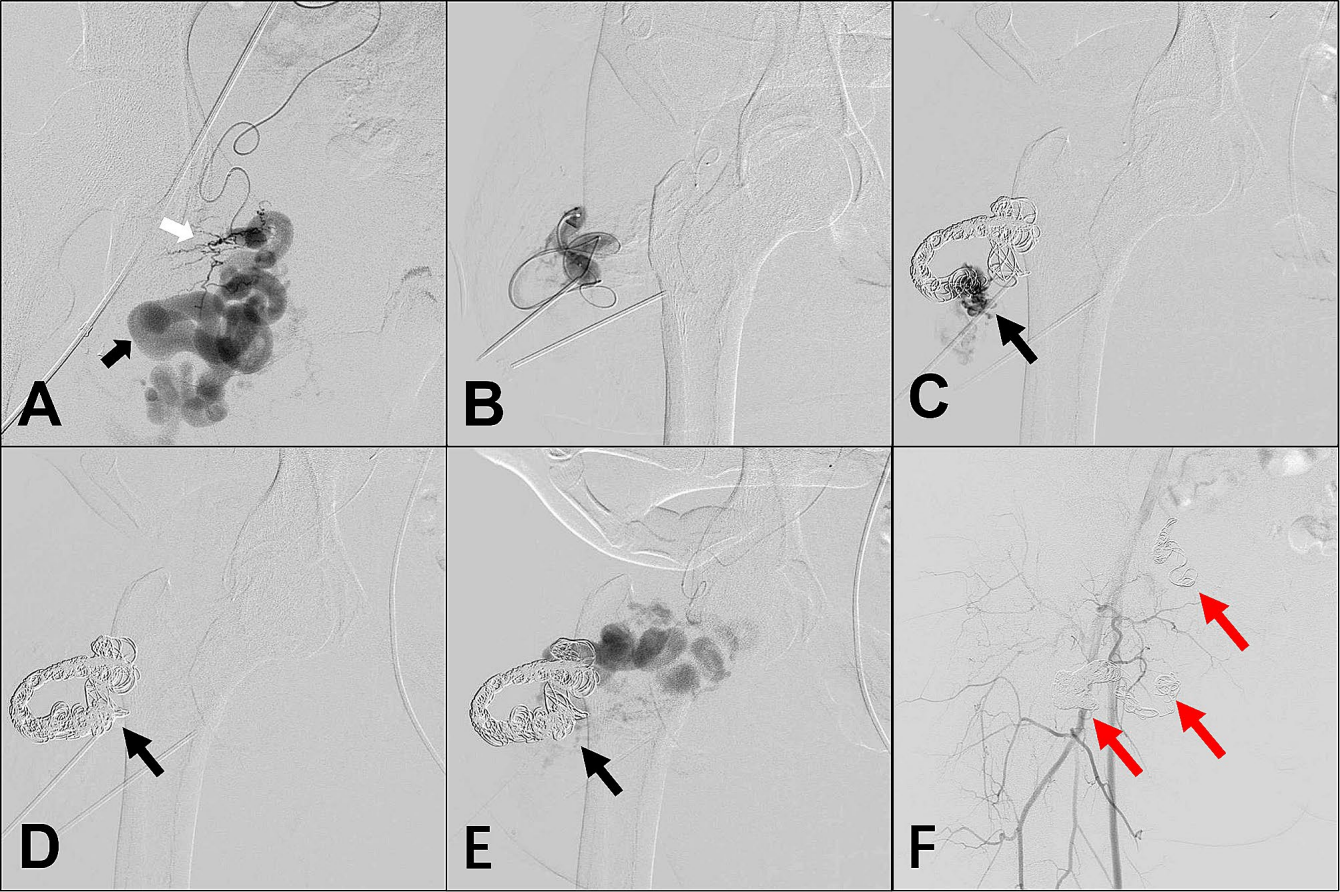
Ethanol embolization assisted with coils treating type IIIb arteriovenous malformations in the buttock (bAVMs). (**a**) Super-selective angiography indicating artery (white arrow), nidus, and dilated vein (black arrow). (**b**) Percutaneous puncture of outflow vein and insertion of microcatheter. (**c**) Configuration of coils and direct-puncture of nidus (black arrow). (**d**) The injection of absolute ethanol within nidus (black arrow). (**e**) Repeat angiography indicated the occlusion of primary nidus (black arrow). (**f**) Final angiography after completed embolization (red arrows: coils in situ)

A baseline angiography (contrast media: Ultravist®, Bayer, Germany) of all the AVMs was performed through the contralateral femoral artery approach to determine the hemodynamic and angioarchitectural features of the AVMs. The dilated vein with the maximum and fastest flow rate in the AVMs lesion was regarded as the dominant outflow vein (DOV) [8]. According to the Yakes classification [14] *Type IIa bAVMs* were treated with the direct puncture of nidus under the guidance of DUS after the baseline angiography. The DOV of the *Type IIb/IIIa/IIIb bAVMs* was identified by super-selective angiography (Fig. 2a). The patients were turned from the supine to the prone position before receiving the puncture procedures. Percutaneous puncture of the DOV was performed with an 18-gauge needle (Cook Medical, Bloomington, IN, USA) under the guidance of DUS. A 2.1-F microcatheter (Asahi, Seto, Japan) was then placed into the DOV through the needle (Fig. 2b). Next, three-dimensional (3D) mechanically detachable coils (Micro Therapeutics Inc., Irvine, CA, USA), followed by pushable coils with attached synthetic fiber (Cook Medical, Bloomington, IN, USA), were deployed through the microcatheter. The coils were injected towards arterial inflow. Next, the direct puncture of the nidus was performed by another 21-gauge butterfly needle or 18-gauge needle under the direction of DUS, and angiography via the needle demonstrated the expected position (Fig. 2c). The ethanol was injected via the second needle introduced into the nidus. It was directed towards the afferent side of the draining vein and delivered in small aliquots based on the amount of contrast medium filling the nidus without the opacification of normal vessels (Fig. 2d). Angiography performed 5–10 min after ethanol injection indicated that the nidus was embolized (Fig. 2e). Repeated ethanol injection was required if the nidus was still present. After the direct puncture procedures, the patients were turned back to the supine position for the final angiography, which demonstrated if residual feeding vessels, nidus, and outflow veins were completely embolized (Fig. 2f). In cases where direct puncture failed because of the deep location of the AVM nidus, trans-arterial embolization of the nidus was performed. A 1.7-F microcatheter was placed coaxially into the nidus, which was confirmed by angiography.

**Fig. 3:**
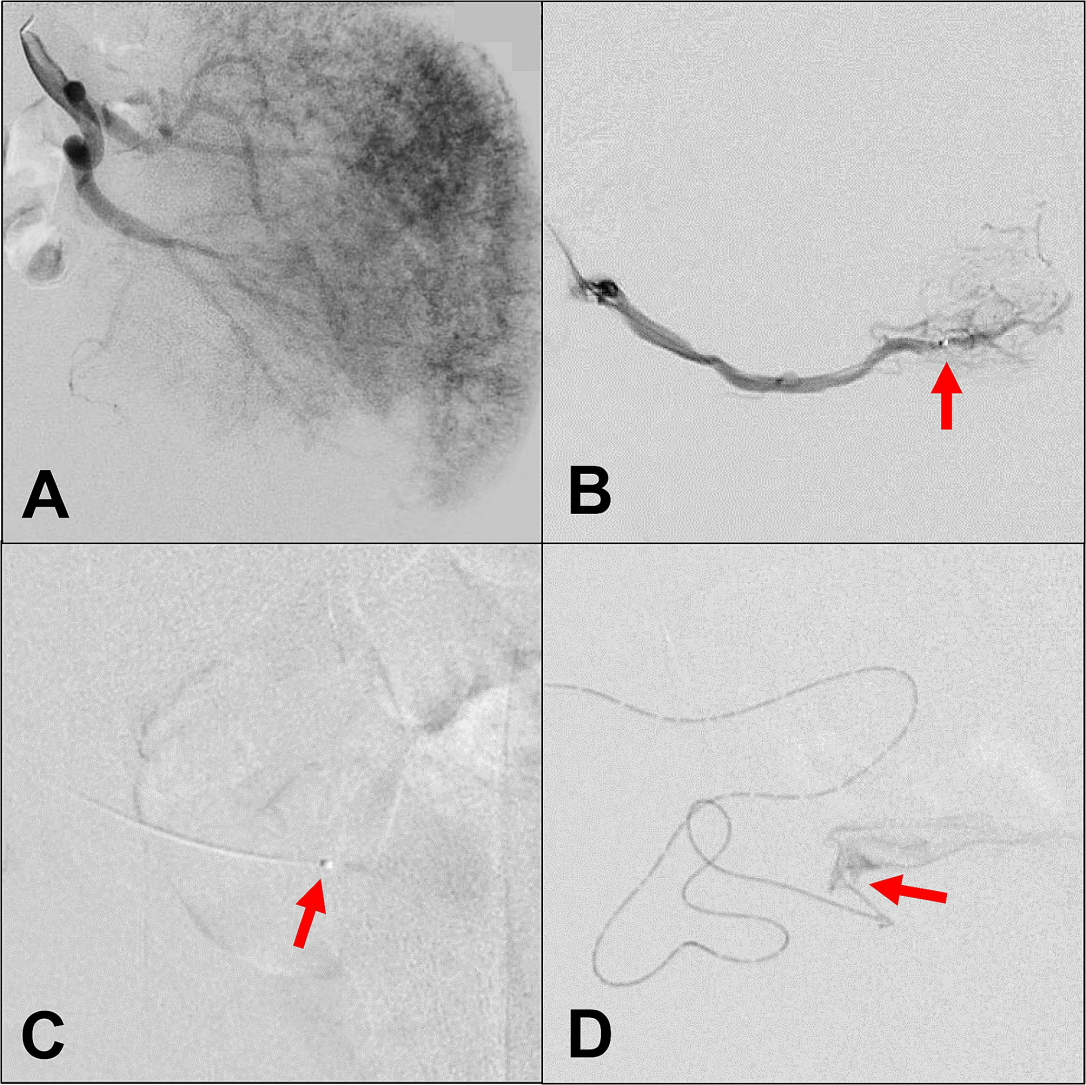
Ethanol embolization treating type IV arteriovenous malformations in the buttock (bAVMs). (**a**) Angiography showed diffused micro-fistulas. (**b**) Super selective angiography by microcatheter. (**c**) Injection of diluted ethanol by microcatheter. (**d**) Super selective angiography of other microarteries. Red arrows: the tip of microcatheter

*Type IV bAVMs* were treated with 50% diluted ethanol, which was made by mixing absolute ethanol with Ultravist® at 1:1 volume. After the baseline angiography of the AVMs (Fig. 3a and Video 1) and arterial super-selection by microcatheter (Fig. 3b), the diluted ethanol was injected trans-arterially (Fig. 3c). Because of the diffuse microfistulas, the remaining microarteries were accessed in order to complete the embolization (Fig. 3d).

The dose of absolute ethanol for a bolus injection was < 0.1 mL/kg, and the total volume in a single embolization session was < 1 mL/kg [15]. Dexamethasone and omeprazole were routinely used to mitigate postoperative swelling and prevent gastrointestinal injury, respectively [15]. The ketorolac tromethamine was administrated routinely to ease postoperative pain.

### Evaluation of clinical outcomes and follow-up results

Patients with extensive AVM lesions were treated in a multistage pattern. One month was set between two embolization procedures to avoid unexpected bleeding episodes, skin ulcer, or necrosis. The postoperative reactions to ethanol embolization include hemoglobinuria, postoperative swelling, bleb formation, and non-purulent exudation. Each patient’s follow-up was conducted as routine clinic visits. Patients were also advised to report or return if they experienced any post-treatment complications: aggravated pain, numbness, local necrosis, and infection.

The therapeutic outcomes were evaluated by noting the amelioration of clinical symptoms and devascularization of bAVMs lesions observed on angiography before and after treatment. The devascularization rate was divided into 0–25%; 26–75%; 76–100% and was evaluated by two independent interventional radiologists with 6 and 10 years of experience, respectively. The senior interventional radiologist with 20 years of experience made the final decision in case of disagreement.

Finally, the therapeutic outcomes were classified as: no response (NR): none of the clinical symptoms disappeared or 0–25% devascularization, partial response (PR): part of clinical symptoms disappeared or 26–75% devascularization, complete response (CR): no residual clinical symptoms, and 76–100% devascularization.

### Statistical analysis

The normality of data was evaluated by the D’Agostino and Pearson test. Data was expressed as the median and inter-quartile range (IQR). The descriptive statistics method was used to calculate each data type’s 95% confidence interval (CI). Two-tailed student’s t-test analyzed the difference between groups. All statistical analyses were performed using SPSS 24.0 (IBM Corp., Armonk, NY, USA) and Prism GraphPad 8.3 (GraphPad Software, San Diego, CA, USA). *P*-value < 0.05 was considered as statistical significance.

## Results

### Baseline data of the bAVMs cohort

The baseline data of 32 bAVMs patients were collected. As shown in Tables 1, 14 were female, and 18 were male, with a mean age of 19.9 years (IQR: 12.8–22.5 years old, 95% CI: 15.47–24.34 years); the 11–20 age group was the largest cohort (15/32; 46.88%). Unilateral involvement was noted in all bAVMs patients. The most common symptom of bAVMs was swelling (28/32; 87.50%), followed by pulsating mass (27/32; 84.38%), elevated skin temperature (20/32; 62.50%), erythema (17/32; 53.13%), bleeding (6/32; 18.75%), pain (5/32; 15.63%), pigmentation (2/32; 6.25%), ulceration (1/32; 3.13%), and bloody stool (melena or blood-streaked stools) (1/32; 3.13%). Thus, 19 out of 32 bAVMs patients (59.38%) were Schöbinger Stage II, and the remaining were Schöbinger Stage III (13/32, 40.63%). Of the 32 patients, 23 (23/32, 71.88%) had received previous therapy, namely 14 prior embolization procedures (14/32; 43.75%), nine surgical excisions (9/32; 28.13%), four radiofrequency ablations (4/32; 12.50%), and four pulsed dye laser therapy (4/32; 12.50%). (see Table 1)

**Table 1:** Baseline data of the patients with arteriovenous malformations in the buttock

Clinical Characteristics	Total Patients (%)	IIa ^1^ (*n* = 7)	IIb ^1^ (*n* = 4)	IIIa ^1^ (*n* = 2)	IIIb ^1^ (*n* = 7)	IV ^1^ (*n* = 12)
**Sex**						
Female	14 (43.75)	3	2	None	3	6
Male	18 (56.25)	4	2	2	4	6
**Age**						
≤ 10	5 (15.63)	None	None	None	2	3
11–20	15 (46.88)	4	2	None	4	5
21–30	7 (21.88)	2	None	2	None	3
> 30	5 (15.63)	1	2	None	1	1
**Localization**						
Left buttock	16 (50)	2	2	1	5	6
Right buttock	16 (50)	5	2	1	2	6
**Previous treatments**						
Present ^2^	23 (71.88)	5	5	1	4	8
Interventional embolization	14 (43.75)	3	2	None	3	6
Pulsed dye laser treatment	4 (12.50)	None	2	None	None	2
Radiofrequency ablation	4 (12.50)	1	None	None	None	3
Surgical excision	9 (28.13)	1	1	1	2	4
Absent	9 (28.13)	2	1	1	3	2
**Symptoms** ^**3**^						
Bleeding	6 (18.75)	3	1	None	None	2
Bloody stool	1 (3.13)	None	1	None	None	None
Erythema	17 (53.13)	4	2	None	4	7
Elevated skin temperature	20 (62.50)	3	2	2	5	8
Swelling	28 (87.50)	5	3	2	7	11
Pain	5 (15.63)	1	None	1	1	2
Pigmentation	2 (6.25)	None	1	None	1	None
Pulsating mass	27 (84.38)	7	4	2	5	9
Ulceration	1 (3.13)	None	None	None	1	None
**Schöbinger Stage** ^**4**^						
I	None	None	None	None	None	None
II	19 (59.38)	3	2	1	5	8
III	13 (40.63)	4	2	1	2	4
IV	None	None	None	None	None	None

(1) According to Yakes Classification. (2) Some of the patients had undergone several previous treatments. (3) Some of the patients had presented multiple symptoms. (4) Schöbinger Stage: Stage I- Pink-bluish stain, warmth, and arteriovenous shunting are revealed by Doppler scanning. Stage II- The description is the same as Stage I, plus enlargement, pulsations, thrill and bruit and tortuous/tense veins. Stage III-Destruction The description is the same as stage II, plus dystrophic skin changes: ulceration, bleeding, persistent pain, or tissue necrosis. Stage IV- The description is the same as stage Ill, plus congestive cardiac failure

### Modality of ethanol embolotherapy

A total of 124 embolization procedures were performed in the cohort, all angiographic data were analyzed based on the Yakes AVMs classification [14]. The most common angiographic type was type IV (12/32; 37.49%), followed by type IIa (7/32; 21.88%) and type IIIb (7/32; 21.88%) and then type IIb (4/32; 12.50%) and type IIIa (2/32; 6.25%). It should be noted that the absolute ethanol consumed per procedure increased in typesIIb and IIIb bAVMs, which possessed multiple outflowing veins. Thirteen patients (type IIb: 4; type IIIa: 2; type IIIb: 7) had undergone coil deployment before ethanol embolization. A more significant number of coils were used per procedure in type III bAVMs than that of type IIbAVMs. (see Table 2) The above results highlighted the management of draining vein in Yake’s Classification.

**Table 2:** Modalities of ethanol embolization of the patients with arteriovenous malformations in the buttock

Yakes Classification	Total patients (%)	No. of procedures (124 in total) ^1^	Absolute ethanol /Procedure (mL)	Coils/procedure * Detachable ^2^ Undetachable ^3^	
Type IIa	7 (21.88)	2–7, mean: 3.57 [2-4.50]	20.94	N/A	N/A
		(1.81–5.31)			
Type IIb	4 (12.50)	3–6, mean: 4.25 [3.75–4.50]	24.53	2.53	7.41
		(2.25–6.25)			
Type IIIa	2 (6.25)	2 and 4, mean: 3.00 N/A	18.50	5.17	14.50
		N/A			
Type IIIb	7 (21.88)	2–9, mean: 4.86 [3.50-6]	25.37	3.85	20.29
		(2.63–7.09)			
Type IV	12 (37.49)	2–7, mean: 3.50 [2.75–4.25]	10.64	N/A	N/A
		(2.54–4.46)			

(1) Each value was followed by [inter-quartile range] and (95% confidence interval). (2) The mechanically detachable coils were 0.018-inch 3D coils (Micro Therapeutics Inc., Irvine, CA, USA), 20 mm in unconstrained diameter and 50 cm in stretched length. (3) The undetachable coils were 0.018-inch 3D Nester coils (Cook, Bloomington, IN, USA) were 8 mm in unconstrained diameter and 14 cm in stretched length

### Therapeutic outcomes of ethanol embolization in bAVMs cohort

Table 3 shows that the mean follow-up period of type IIa, IIb, IIIa, IIIb, and IV bAVMs patients was 51, 28.35, 51, 41, and 24.33 months, respectively. Concerning angiographic outcomes, only one patient (type IV) achieved less than 25% devascularization rate; 13 patients exhibited 26–75% devascularization (40.63%); 18 patients revealed 76–100% devascularization (56.25%). After ethanol embolization, clinical symptom improvement was found in 23 of 27 who presented with a pulsating mass (85.19%), 17 of 20 with abnormal local skin temperature (85%), 5 of 6 with bleeding (83.33%), and 5 of 5 patients with pain (100%). Finally, 12 out of 13 patients (92.31%) reduced from Schöbinger Stage III to a lower grade. (Tables 1 and 3)

**Table 3:** Clinical and angiographic outcomes of the patients with arteriovenous malformations in the buttock

Outcomes	Total Patients (%)	IIa ^1^ (*n* = 7)	IIb ^1^ (*n* = 4)	IIIa ^1^ (*n* = 2)	IIIb ^1^ (*n* = 7)	IV ^1^ (*n* = 12)
**Mean follow-up (months)**		51	28.35	51	41	24.33
**Residual Symptoms** ^**2**^						
Bleeding	1 (3.13)	1	None	None	None	None
Bloody stool	None	None	None	None	None	None
Erythema	9 (28.13)	1	1	None	3	4
Elevated skin temperature	3 (9.38)	None	1	None	1	1
Swelling	17 (53.13)	2	1	2	3	9
Pain	None	None	None	None	None	None
Pigmentation	1 (3.13)	None	1	None	None	None
Pulsating mass	4 (12.50)	2	1	None	None	1
Ulceration	None	None	None	None	None	None
**Devascularization rate**						
0–25%	1 (3.13)	None	None	None	None	1
26–75%	13 (49.63)	3	2	None	2	6
76–100%	18 (56.25)	4	2	2	5	5
**Therapeutic Outcome**						
No response	1 (3.13)	None	None	None	None	1
Partial response	21 (65.63)	3	3	2	4	9
Complete response	10 (31.25)	4	1	None	3	2
**Schöbinger Stage** ^**3**^						
I	1 (3.13)	None	None	None	1	None
II	30 (93.75)	6	4	2	6	12
III	1 (3.13)	1	None	None	None	None
IV	None	None	None	None	None	None
**Postoperative reactions & complications**						
Hemoglobinuria	6 (18.75)	3	1	None	2	None
Postoperative swelling	32 (100)	7	4	2	7	12
Bleb	14 (43.75)	3	3	2	2	4
Non-purulent exudation	12 (37.50)	1	3	1	2	5
Necrosis	1 (3.13)	None	None	None	None	1

(1) According to Yakes Classification for arteriovenous malformations. (2) Some of the patients had multiple symptoms. (3) Schöbinger Stage: Stage I- Pink-bluish stain, warmth, and arteriovenous shunting are revealed by Doppler scanning. Stage II- The description is the same as Stage I, plus enlargement, pulsations, thrill and bruit and tortuous/tense veins. Stage III-Destruction The description is the same as stage II, plus dystrophic skin changes: ulceration, bleeding, persistent pain, or tissue necrosis. Stage IV- The description is the same as stage Ill, plus congestive cardiac failure

**Fig. 4:**
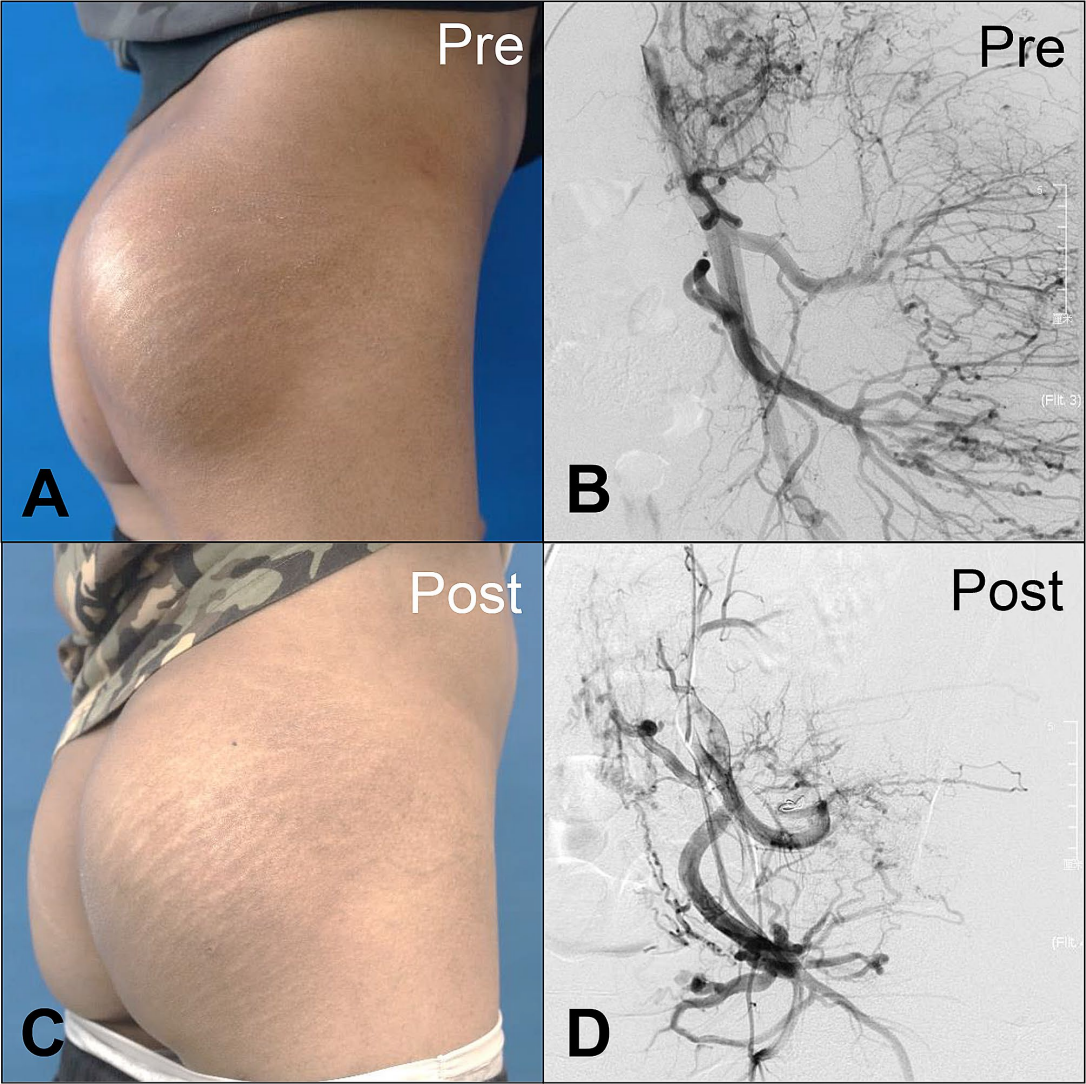
Representative case of Yakes type IIa arteriovenous malformations in the buttock (bAVMs). Patient 20 had a pulsating mass in the right buttock, accompanied by local hypertrophy (Schöbinger Stage II) (**a**, **b**). The patient achieved > 90% devascularization after the operation. The therapeutic effect was stable in this patient for up to nine years (**c**, **d**)

**Fig. 5:**
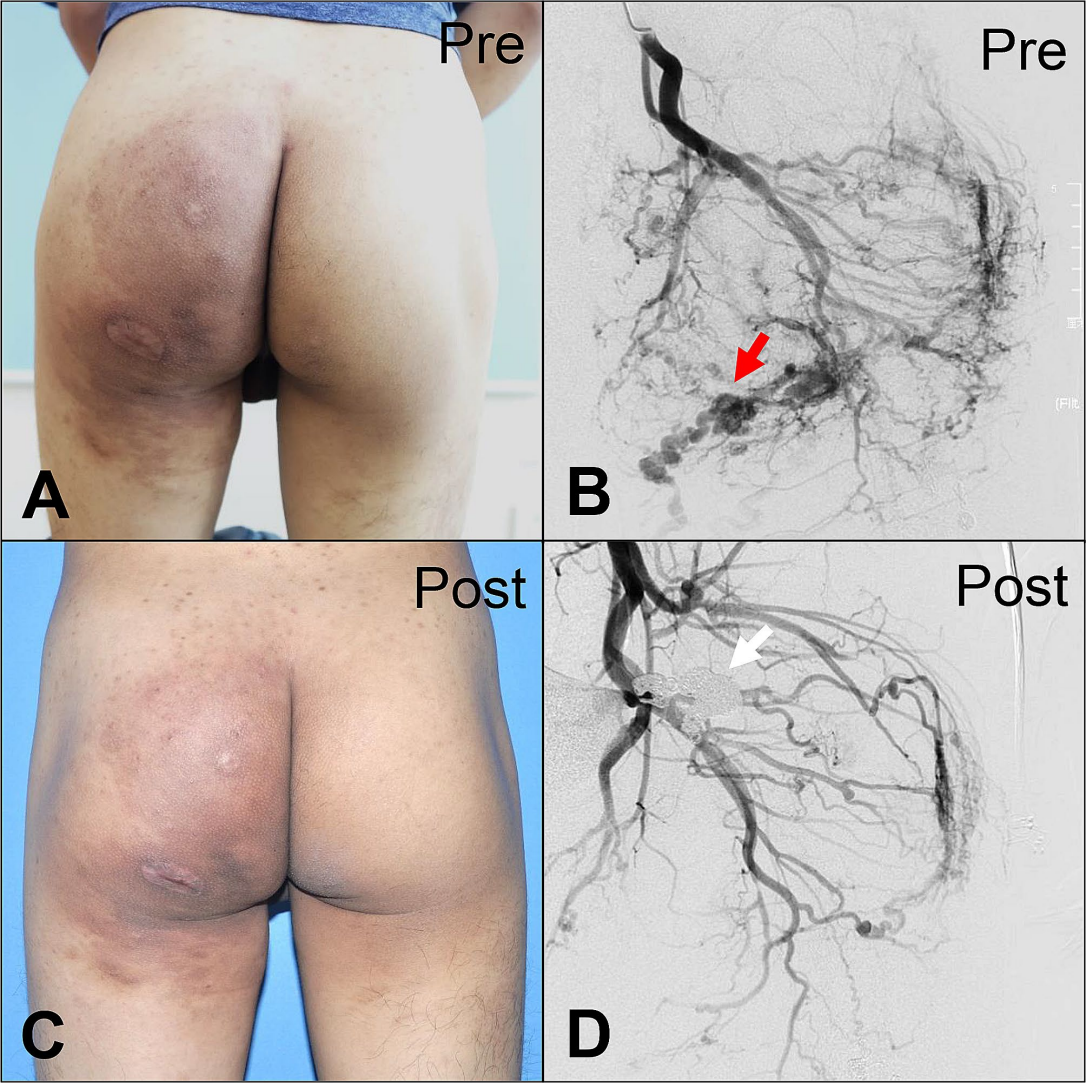
Representative case of Yakes type IIb arteriovenous malformations in the buttock (bAVMs). Patient 13 presented with local swelling, elevated skin temperature, pigmentation, pulsating mass, and bloody stool (Schobinger stage III) (**a**, **b**). The patient underwent six procedures of ethanol embolization. At 5-month follow-up, the primary lesion achieved nearly 75% vascular occlusion, accompanied by significant reduction in size (**c**, **d**). Red arrow: aneurysmal outflow vein. White arrow: coils in situ

**Fig. 6:**
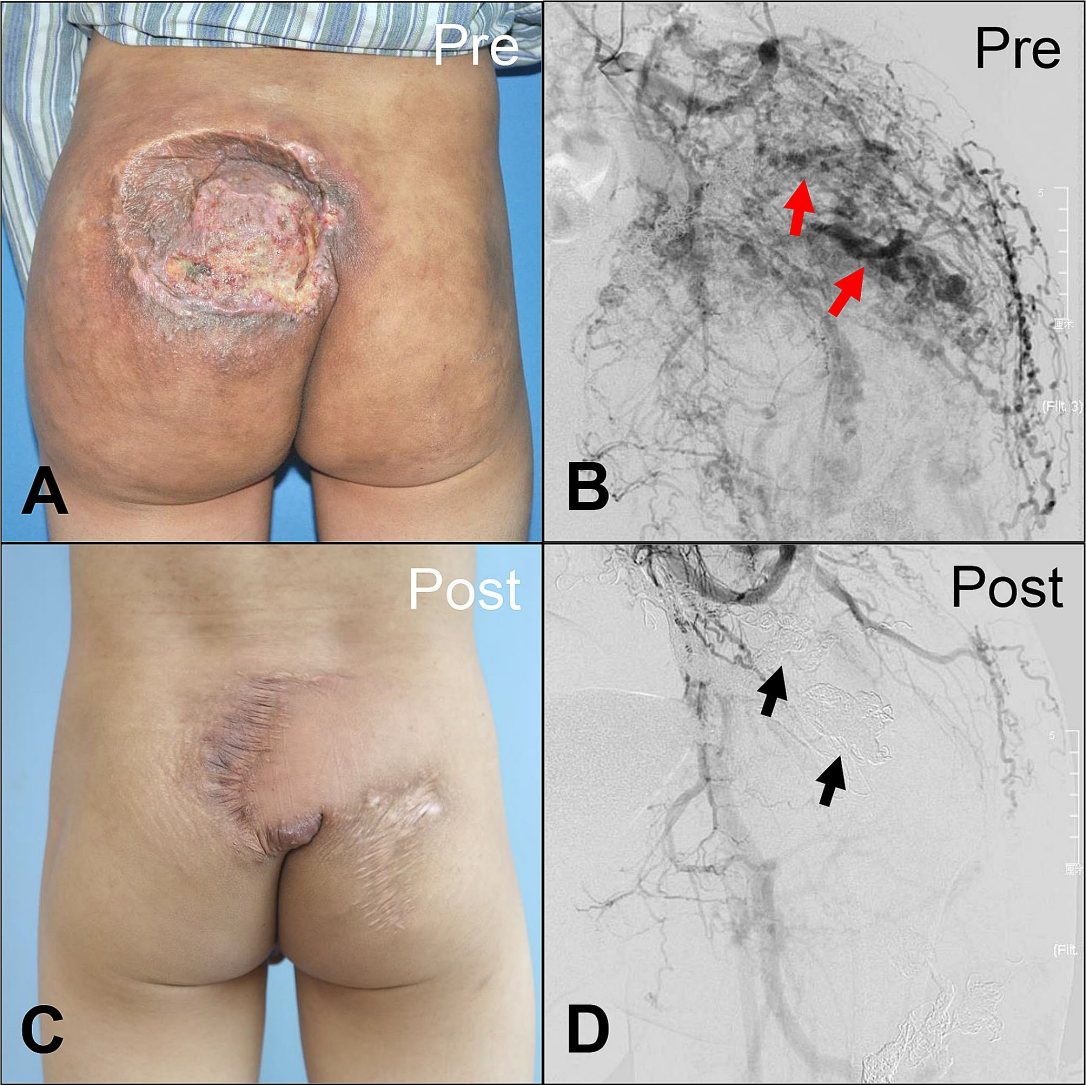
Representative case of Yakes type IIIb arteriovenous malformations in the buttock (bAVMs). Patient 5 presented the typical manifestation of Schöbinger stage III bAVMs, including severe ulceration and pain (**a**, **b**). The patient underwent a total of nine episodes of ethanol embolization and obtained > 90% devascularization. Thereafter, a debridement was performed to promote wound healing, and the defect was repaired simultaneously using a regional rotation flap (**c**, **d**). Red arrows: dilated outflow veins. Black arrows: coils in situ

Taken together, ten patients exhibited a CR (10/32; 31.25%), 21 patients showed a PR (21/32; 65.63%), and only one patient demonstrated NR (1/32; 3.13%) (see Table 3). Pre- and post-embolization images of representative cases were shown in Figs. 4, 5 and 6.

### Postoperative reactions and complications

As for ethanol embolization-related reactions, hemoglobinuria was found after sixteen procedures (16/124, 12.90%) among six patients (6/32, 18.75%) when the dose of absolute ethanol was > 30 mL per procedure. The transient hemoglobinuria presented with visible dark urine and was treated by increasing fluid replacement for six hours. Postoperative swelling happened in the wake of each ethanol embolization procedure. Cutaneous blebs occurred in 14 out of 32 patients (43.75%); whereas non-purulent exudation happened in 12 patients (37.50%). (see Table 3)

Regarding postoperative complications, one bAVMs patient with extensive erythema skin discoloration suffered from embolization-related local necrosis (1/32, 3.13%) after two entire embolization (2/124, 1.61%). Neither paranesthesia nor infection was observed in follow-ups. (Table 3)

## Discussion

Reports about bAVMs remain scarce, the present study retrospectively analyzed a cohort of 32 patients with bAVMs who received curative ethanol embolization. During regular follow-ups, bAVMs patients obtained a favorable devascularization rate and improved clinical symptoms.

Peripheral AVMs are complex lesions and can be very aggressive locally. They can cross tissue boundaries and even penetrate cortical bone [1]. Endovascular embolization is particularly effective when treating peripheral AVMs, especially when done using the guidance of the Yakes classification. Griauzde et al. [16] reported a cohort of AVMs in the head and neck region. After complete embolization, the Schöbinger stage was downgraded in all patients. Arteriovenous shunt eradication of > 90% was achieved in most patients. Yakes type IV lesions required more treatment sessions, and shunt eradication was higher in the Yakes II and III groups. Bouwman, et al. [17] conducted a clinical study on peripheral AVMs, including the head and neck, thorax, abdomen, and upper and lower extremities. By using ethanol and coils, embolization resulted in 70–100% devascularization in most patients, with few major complications. Their studies support the rationality and practicability of Yakes classification in peripheral AVMs’ intervention.

Anatomically, the buttock comprises thick skin, well-developed superficial fascia, and dense adipose tissue, dubbed “fat pad”. All these distinguishing characteristics enable the buttock region to withstand intense and persistent pressure [18]. Our study found that the bAVMs are always insidious at an early age; whereas the primary bAVMs lesion had already developed into an extensive form when the patient reported subjective symptoms (see Table 1) The feeding artery of gluteal tissue is the internal iliac artery, which then divides into the superior gluteal artery, inferior gluteal artery, and internal pudendal artery [19]. Thus, the angiography through the internal and external iliac artery provides a comprehensive morphology of bAVMs lesions (Figs. 4, 5 and 6).

The available therapeutic options for AVMs include surgical excision, endovascular embolization, or a combination of these procedures [17, 20]. The successful experiences of endovascular embolization have further made it a first-line therapeutic strategy in treating advanced non-resectable disease [10]. Many types of embolic agents are available, including ethanol, Onyx®, n-butyl cyanoacrylate (NBCA), and coils. Liquid embolic agents such as NBCA and Onyx® have been widely used in treating brain AVMs although complications have been reported [21–24]. However, clinical records show that these reagents cause recurrence and complications when it comes to peripheral AVMs [25]. Unlike NBCA, Onyx® has low viscosity and delayed precipitation properties, providing better embolization control and effects. Despite these advantages, due to its black color, subcutaneous exudation during superficial embolization can cause skin discoloration [26]. In present study, many of the patients with bAVMs had received the previous treatments (Table 1), most of which were embolized with NBCA or Onyx®. Ethanol, a liquid embolic agent, has been widely applied in vascular malformations since its introduction in treating renal arteriovenous fistula [27]. Unlike Onyx and NBCA, ethanol leads to protein denaturation [28]. Due to dehydration, ethanol can destroy vascular endothelial cells and cause intraluminal thrombosis, further achieving positive therapeutic effects in bAVMs [29].

For extremely high-flow AVMs, the abnormal hemodynamic conditions should be controlled not only to relieve the unusual impact force induced by high-pressure arterial blood on the venous wall but also to prolong the exposure time of vascular endothelium to high-concentration ethanol [10]. According to the Yakes AVMs classification, coils are recommended in the management of type IIb, type IIIa, and type IIIb AVMs, all of which possess the “aneurysmal” vein in angioarchitecture [16].

For bAVMs without an “aneurysmal” vein, we conducted embolization using direct-puncture and trans-arterial approaches. A direct puncture technique was first attempted in bAVMs and was later proven to be effective for targeting the nidus or key lesions of AVMs. However, in some AVMs, especially in the type IV AVMs, micro-fistulas caused by the shunts between arterioles and venules are ubiquitous (Fig. 3a). Given that micro-fistulas with minute lumen are always hard to pierce by the puncture needle, embolization by direct blind puncture might lead to the interstitial injection of ethanol and further result in necrosis of superficial tissue. Therefore, trans-arterial ethanol embolization was performed to compensate for the direct-puncture approach, making the ethanol flow along the abnormal angioarchitecture, allowing abundant destruction of the vascular endothelium [30].

Given the complexity of managing peripheral AVMs, they are better handled by a multidisciplinary vascular team equipped to offer a multimodal care approach [31]. Firstly, at diagnosis, DUS combined with contrasted-enhanced CT is able to provide the pathomorphology of vasculature and perivascular tissue. Secondly, an organized endovascular embolization should be the first line of treatment. It is noteworthy that surgical therapy is not recommended until the AVMs lesion is embolized sufficiently [7]. As we have shown, the bAVMs patient who suffered from severe ulceration, surgical resection and reconstruction was essential to clear out necrotic tissue and further repair the defect (Fig. 6). Recently, hyperbaric oxygen therapy has shown promising effects in treating chronic ulcerations caused by vasculitis [32]. In our center, hyperbaric oxygen therapy has been performed on rescuing Schrödinger stage III AVMs patients who had severe ulceration. Finally, AVMs are a series of high-flow vascular disorders caused by predominantly somatic mutations, although some are germline, as in capillary malformation-arteriovenous malformation [33]., MEK/MAPKK (Mitogen-Activated Protein Kinase Kinase) inhibitors targeted against somatic MAP2K1 mutations are being developed to be used as primary or adjuvant therapy in managing peripheral AVMs [34]. All in all, excellent coordination and multidisciplinary cooperation are vital for the treatment of peripheral AVMs.

The present work has limitations. Firstly, selection bias is hard to avoid in a retrospective study, and the AVMs were heterogeneous. Secondly, a wide range of enrollment and the small sample size limited the validity of the results, though some are promising.

## Conclusion

In conclusion, ethanol embolization seems to be safe and effective in treating bAVMs. Mechanical embolization by coils also plays a pivotal role in treating the lesion with extremely high blood flow. The present work demonstrated the pattern of bAVMs related to the buttock’s anatomy, and further highlighted the bAVMs’ management based on the Yakes classification.

## Data Availability

All data generated or analyzed during this study are included in this published article.
